# Brachytherapy for cervix cancer: low-dose rate or high-dose rate brachytherapy – a meta-analysis of clinical trials

**DOI:** 10.1186/1756-9966-28-47

**Published:** 2009-04-05

**Authors:** Gustavo A Viani, Gustavo B Manta, Eduardo J Stefano, Ligia I de Fendi

**Affiliations:** 1 Radiation Oncology Department at Marilia School of Medicine, São Paulo, Brazil; 2 Medical Student at Marília School of Medicine, São Paulo, Brazil; 3 Department of Ophthalmology at Marília medicine school, São Paulo, Brazil

## Abstract

**Background:**

The literature supporting high-dose rate brachytherapy (HDR) in the treatment of cervical carcinoma derives primarily from retrospective series. However, controversy still persists regarding the efficacy and safety of HDR brachytherapy compared to low-dose rate (LDR) brachytherapy, in particular, due to inadequate tumor coverage for stage III patients. Whether LDR or HDR brachytherapy produces better results for these patients in terms of survival rate, local control rate and the treatment complications remain controversial.

**Methods:**

A meta-analysis of RCT was performed comparing LDR to HDR brachytherapy for cervix cancer treated for radiotherapy alone. The MEDLINE, EMBASE, CANCERLIT and Cochrane Library databases, as well as abstracts published in the annual proceedings were systematically searched. We assessed methodological quality for each outcome by grading the quality of evidence using the Grading of Recommendations Assessment, Development and Evaluation (GRADE) methodology. We used "recommend" for strong recommendations, and "suggest" for weak recommendations.

**Results:**

Pooled results from five randomized trials (2,065 patients) of HDR brachytherapy in cervix cancer showed no significant increase of mortality (p = 0.52), local recurrence (p = 0.68), or late complications (rectal; p = 0.7, bladder; p = 0.95 or small intestine; p = 0.06) rates as compared to LDR brachytherapy. In the subgroup analysis no difference was observed for overall mortality and local recurrence in patients with clinical stages I, II and III. The quality of evidence was low for mortality and local recurrence in patients with clinical stage I, and moderate for other clinical stages.

**Conclusion:**

Our meta-analysis shows that there are no differences between HDR and LDR for overall survival, local recurrence and late complications for clinical stages I, II and III. By means of the GRADE system, we recommend the use of HDR for all clinical stages of cervix cancer.

## Introduction

Intracavitary radiation in the form of low-dose rate (LDR) brachytherapy has been in use for the treatment of cervical cancer for nearly a century, although the method has been greatly refined. High-dose rate (HDR) brachytherapy for carcinoma of the cervix has been in use for over 30 years. LDR is defined as a dose of 0.4–2 Gray (Gy)/h, and HDR is defined as a dose of >12 Gy/h [1]. HDR is widely used throughout Asia and Europe, and its use is steadily increasing in North and South Americas [2]. The Patterns of Care Studies show that, in the United States, the use of HDR for the treatment of cervical cancer increased from 9% during 1992–1994 to 16% during 1996–1999, although this increase did not reach significance[3].

LDR techniques were developed in an era when remote afterloading technology was unavailable, and remote afterloading techniques were developed due to concerns related to radiation exposure to health care workers. In more recent years, new technology has allowed remote afterloading brachytherapy to be given at LDR. The use of HDR brachytherapy is the result of technological development in the manufacture of high-intensity radioactive sources, sophisticated computerized remote afterloading devices, and treatment planning software [4]. Several advantages of HDR brachytherapy, including rigid immobilization, outpatient treatment, patient convenience, accuracy of source and applicator positioning, individualized treatment with source optimization, and complete radiation protection for personnel have been claimed [5-7].

There are nearly three decades of experience comparing HDR to LDR brachytherapy in the treatment of cervical carcinoma. The literature supporting HDR brachytherapy in the treatment of cervical carcinoma derives primarily from retrospective series [8-14]. However, controversy still persists regarding the efficacy and safety of HDR brachytherapy compared to low-dose rate (LDR) brachytherapy [2-4,15]. In particular, due to inadequate tumor coverage for stage III patients, whether LDR or HDR brachytherapy produces better results for this patients in terms of survival rate, local control rate and treatment complications remain controversial.

So, the goal of this study was to conduct a meta-analysis to compare the efficacy and safety of HDR and LDR brachytherapy in patients with cervix cancer. In addition to that, we found it appropriate to build the recommendations for the use of HDR based on the GRADE system.

## Materials and methods

Criteria for considering studies for this review included the following:

### Studies

RCTs or overviews of RCTs comparing LDR brachytherapy to HDR brachytherapy in patients with cervical carcinoma treated with radiotherapy alone or combined to chemotherapy, which were fully published in journals and those identified from other sources (abstracts and proceedings of relevant scientific meetings, and contact with investigators).

### Study population

Patients with histologically confirmed cervical cancer and at least 18 years of age.

### Interventions

Trials that compared HDR brachytherapy to LDR brachytherapy following pelvic radiotherapy.

### Outcome measures

Overall mortality, local recurrence and treatment complications. The databases MEDLINE (Ovid) (1996–May, 2007), CANCERLIT (Ovid) (1996–March 2007) and the Cochrane Library (Issue 2, 2007) were searched for trials using the terms: 'low-dose rate' (Medical Subject Heading [MeSH]), 'high-dose rate' (text words), 'intracavitary radiotherapy' (text word), 'brachytherapy' (text word) and 'cervical cancer' or 'cervix cancer' (MeSH and text word). These terms were then combined with the search terms for the following study designs: practice guidelines, systematic reviews or meta-analyses, reviews, randomized controlled trials and controlled clinical trials. In addition, the Physician Data Query (PDQ) clinical trials database on the Internet , and the proceedings of the 1997–2007 annual meetings of the American Society of Clinical Oncology (ASCO) and the American Society of Radiation Therapist (ASTRO) were searched for reports of new or on-going trials. Relevant articles and abstracts were selected and reviewed by two methodologists, and the reference lists from these sources were searched for additional trials. Randomized trials identified by the search were assessed to determine whether they met the inclusion criteria. They were assessed by two independent reviewers (V GA., S EJ.). Discrepancies were resolved by a third reviewer (F LI.).

### Analysis of the review

We used two techniques to calculate the pooled odds ratio (OR) estimates: the Mantel-Haenszel method [16] assuming a fixed-effects model and the Der Simonian-Laird method [17] assuming a random-effects model. The fixed-effects model leads to valid inferences about the specific studies that have been assembled, and the random-effects model assumes that the particular study samples were drawn from a larger universe of possible studies and leads to inferences about all studies in the hypothetical population of studies. The random-effects approach often leads to wider confidential intervals (CIs). In the absence of heterogeneity, the fixed and random-effects models provide similar results. ORs less than unity indicated a treatment effect that favored the study agent. Pooled, weighted ORs and their respective 95% CIs were then estimated separately per each outcome for each meta-analysis.

In the RCTs that have reported the severity of complications classified according to the Radiation Therapy Oncology Group (RTOG) or other score systems were combined when possible. Subgroup analyses for each outcome were performed by recalculating the ORs and 95% CIs, based on the clinical stage of the disease. We evaluated heterogeneity across trials using the I^2 ^statistics, which describes the percentage of total variation across studies that are due to heterogeneity rather than chance [18]. The interpretation of I^2 ^depends on the magnitude and direction of effects, as well as the strength of evidence for heterogeneity (e.g. P value from the chi-squared test, or a confidence interval for I^2^) [19]. We used the following classification based on the value of I^2 ^[17,18]: 0–30 = low; 30–60 = moderate and worthy of investigation; 60–90 = severe and worthy of understanding; 90–100 = allowing aggregation only with major caution. Publication bias is a common concern in meta-analysis, which is related to the tendency of journals to favor the publication of large and positive studies. Quality of the evidence has been assessed using the grade four-category system (high, moderate, low and very low quality) (Table 1). Factors that are considered in classifying evidence are: the study design and rigor of its execution, the consistency of results and how well the evidence can be directly applied to patients, interventions, outcomes and comparator. Other important factors are whether the data are sparse or imprecise and whether there is potential for reporting bias. Using this approach, assessments of the quality of evidence for each important outcome take into account the study design, limitations of the studies, consistency of the evidence across studies, the directness of the evidence, and the precision of the estimate [20,21].

**Table 1:** Quality of the quality evidence, definitions and underlying methodology

**Grade**	**Definition**	**Underlying Methodology**
High	Further research is very unlikely to change our confidence in the estimate of effect	RCT or meta-analysis

Moderate	Further research is likely to have an important impact on our confidence in the estimate of effect and may change the estimate	Downgraded RCTs or upgraded observational studies

Low	have an important impact on our confidence in the estimate of effect an its likely to change the estimate	Well-done observational studies with control groups

Very low	Any estimate of effect is very uncertain	Others (e.g., case reports or case series)

For each intervention considered, we formulated a consensus recommendation based on our judgments, regarding the balance between the benefits, harms (adverse effects), costs, and values and preferences of the intervention. Then, recommendations have been classified as "strong" or "weak." (Table 2)

**Table 2:** Strength of recommendations and implication to quality of evidence.

**Recommendation or statement**	**Description in GRADE approach**	**Interpretation**
Strong recommendation	We recommend (should)	1. Most individuals should receive the intervention, assuming that they have been informed about and have understood its benefits, harms and burden.
		2. The recommendation could unequivocally be used for policy making.

Weak recommendation	We suggest (might)	1. Uncertainty about the relative importance of the benefits and downsides to those affected, or differences in how important they are to different people, which could affect the balance between the benefits versus harms and burden
		2. Doubt about the recommendation could be use for policy making

We chose a commonly used method for detecting publication bias, which is a graphical plot of estimates of the odds ratios from the individual studies versus the inverse of their variances, which is commonly referred to as a "funnel plot." The analyses were performed using comprehensive meta-analysis software (Revman 5.0).

## Results

**Figure 1:**
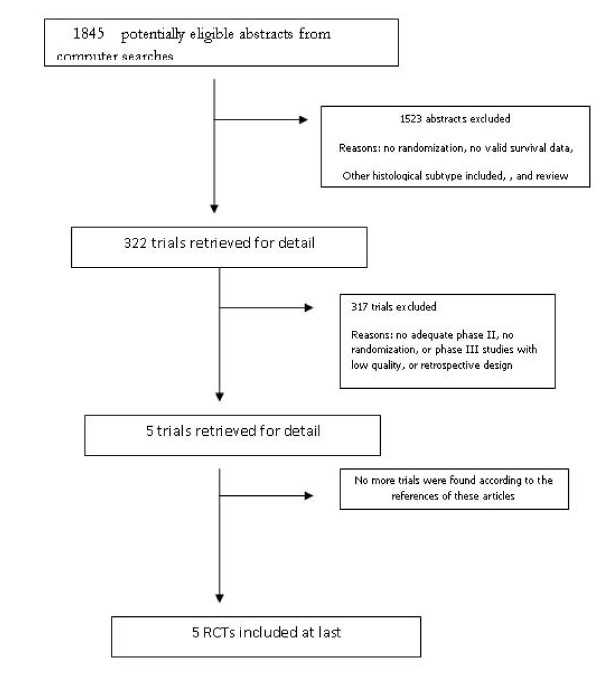
**Flowchart according to QUOROM statement criteria, informing the reason of some trials to be excluded**.

**Figure 2:**
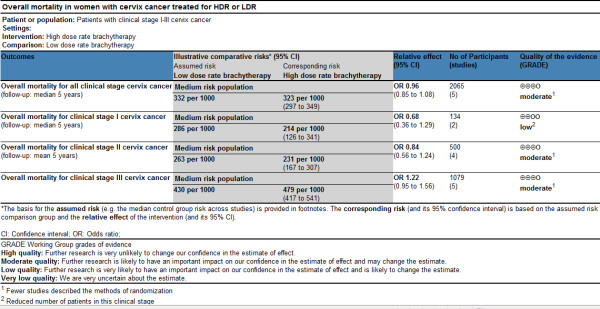
**Summary of findings (SoF) table using GRADE methodology for overall mortality**.

**Figure 3:**
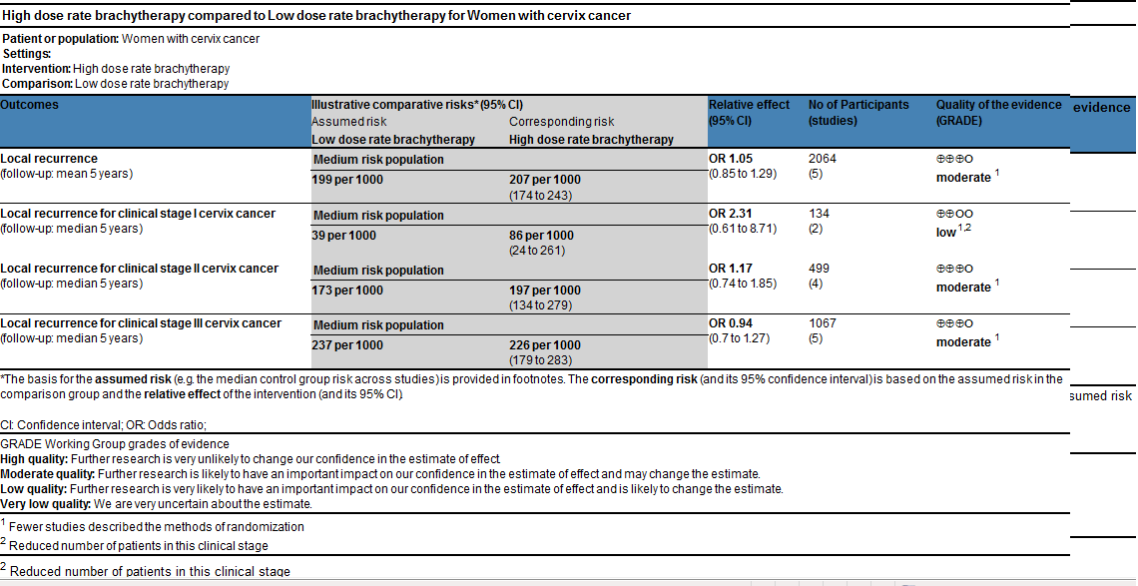
**Summary of findings (SoF) table using GRADE methodology for local recurrence**.

The two trial assessors agreed on the selection of five RCTs. The Quorum flow diagram illustrates the main reasons for trial exclusion (Figure 1). The overall sample included 2,145 patients in 5 RCTs comparing LDR to HDR [22-26]. The published studies are described in Table 3 and the quality of studies is described in Figure 2 and Figure 3

**Table 3:** Characteristics of clinical trials

**Year**	**Study**	**Patients**	**Fraction of LDR** **(Gy/fraction)**	**Fraction of HDR** **(Gy/Fraction)**	**Pelvic RT Dose** **(Gy)**	**Clinical stage**	
						
						**LDR**	**HDR**
2004	Lertsanguansinchai	237	25–35/2	15–16.6/2	40–50	IB-5	IB-7
						IIA-2	IIA-1
						IIB-61	IIB-64
						IIIB-41	IIIB-40

2002	Hareyama	132	IIA-50/4	IIA-29,5/4	30–40	II-26	II-22
			IIB-40/3	IIB-23,3/3 or 4		III-39	III-45
			III-30/3	III-17,3/3 or 2			

1993	Teshima	430	I-56/2	I-28/4	16–20	I-28	I-32
			II-57/2	II-30/4		II-61	II-80
			III-58/2	III-29/3		III-82	III-147

1994	Patel	482	I-II>3 cm-75/2	I-II>3 cm-38/2	35–40	I-39	I-35
			I-II<3 cm-35/1	I-II<3 cm-18/2		II-93	II-90
			III-35/1	III-18/2		III-114	III-111

2006	Shrivastava	800	I and II-60/2	I and II-35/5	40/20	I II-200	I II-200
			III-30/1	III-21/3		III-200	III-200

### Methodological quality of included studies

Following the GRADE system, the study design for all trials included in the review of evidence for HDR and LDR was randomized controlled trial, which is scored as a high type of evidence. As requested from the methodology of GRADE, study quality was also assessed by reviewing whether the studies had limitations or flaws. The following limitations were noted, leading frequently to a decrease in the quality of evidence: methods of randomization were not clearly reported, allocation concealment was not reported or unclear, none of trials were blinded, incomplete descriptions of withdrawals and dropouts were reported, analyses were based on the per protocol or completer population and not on the intention-to-treat population and none of studies reported a priori sample size calculations. The percentage follow-up ranged from 89% to 100%. None of the studies was interrupted early for benefit. The methodological quality varied by outcome. It was low for mortality and local recurrence in clinical stage I and moderate for other outcomes (Figure 2, Figure 3). Figure 2 and Figure 3 also provide the absolute reductions in the risks of different outcomes for a number of illustrative baseline risks, including medium baseline risks.

### Overall mortality

**Figure 4:**
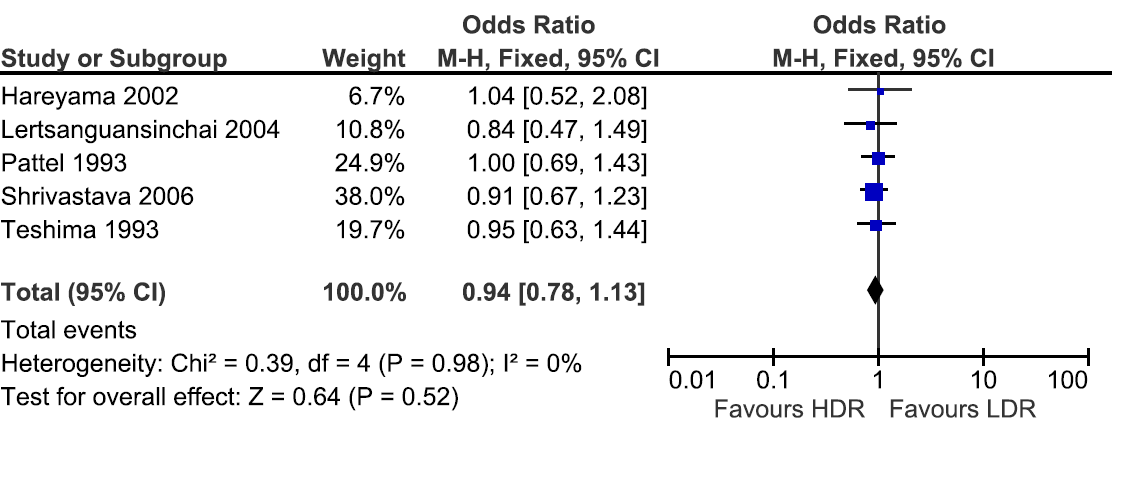
**Overal mortality for all clinical stages in cervix cancer**.

Five studies reported overall mortality as one of the outcomes. Altogether, the analyses included 5 trials with 2,065 patients. The overall mortality rates were not decreased for LDR arm (340/997 = 34.1%) compared to HDR arms (375/1068 = 35.1%). The overall odds ratio (OR = 0.94, CI 95% -0.78, 1.13) suggests that there is no difference between LDR arms and HDR arms in terms of overall mortality rate with p value 0.52, as demonstrated in Figure 4. The test for heterogeneity was not statistically significant with p value 0.98, which indicates that the pooling of the data was valid. In the subgroup analysis there was no difference for overall survival among different clinical stages I, II and III, as demonstrated in Table 4.

**Table 4:** LDR versus HDR for overall mortality, local recurrence and late complications

**Overall mortality**							
**Stage**	**Number of studies**	**Total patients**	**Patients**/**events** **HDR**	**Patients**/**events** **LDR**	**OR**	**CI95%**	**P value**

I	2	134	19/67	13/67	0.68	0.36–1.29	0.23

II	4	500	75/257	62/243	0.84	0.56–1.24	0.38

III	5	1079	238/572	228/507	1.22	0.95–1.56	0.11

**Local recurrence**							

**Stage**	**Number of studies**	**Total patients**	**Patients**/**events** **HDR**	**Patients**/**events** **LDR**	**OR**	**CI95%**	**P value**

I	2	134	7/67	3/67	2.31	0.61–8.71	0.22

II	4	500	45/257	34/243	1.17	0.74–1.85	0.51

III	5	1079	143/572	138/507	0.94	0.70–1.27	0.70

**Grade 3 or 4 rectal complication**							

	**Number of studies**	**Total patients**	**Patients/events** **HDR**	**Patients/events** **LDR**	**OR**	**CI95%**	**P value**

	5	2065	27/1068	27/997	0.9	0.52–1.56	0.7

**Grade 3 or 4 bladder complication**							

	**Number of studies**	**Total patients**	**Patients**/**events** **HDR**	**Patients**/**events** **LDR**	**OR**	**CI95%**	**P value**

	5	2065	17/1068	16/997	0.98	0.49–1.96	0.95

**Grade 3 or 4 small intestine complication**							

	**Number of studies**	**Total patients**	**Patients**/**events** **HDR**	**Patients**/**events** **LDR**	**OR**	**CI95%**	**P value**

	3	783	13/432	3/351	3.15	0.9–10.37	0.06

### Local recurrence

**Figure 5:**
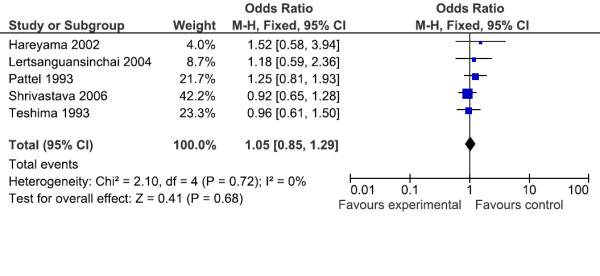
**Local recurrence for all clinical stages in cervix cancer**.

Five trials reported on local control. There was no significant difference for local recurrence among those patients receiving LDR brachytherapy and HDR brachytherapy (OR = 1.05(CI 95% 0.85–1.29), as Figure 5. The test for heterogeneity was not statistically significant with p value 068, which indicates that the pooling of the data was valid. In the subgroup analysis there was no difference for overall survival among different clinical stages I, II and III, as demonstrated in Table 4.

### Grade 3 or 4 Rectal, Bladder or Small Intestine complications

Five trials evaluated rectal or bladder complications. For grade 3 or 4 rectal and bladder complication, there was no significant difference between HDR and LDR, as demonstrated in Table 4. Only 3 studies reported the small intestinal complications as one of its outcomes. No significant difference was observed between the treatment arms, considering grade 3 or 4 complication, as showed in Table 4.

## Discussion

Approximately 11,070 women are diagnosed with cervical cancer annually in the US, resulting in 3,870 deaths [27]. This represents 0.13 percent of all cancer deaths in women. Despite this, and the promise of newly developed cervical carcinoma vaccines [28], cervical carcinoma is still the third largest cancer killer of women world-wide, causing 274,000 deaths in 2002 [29]. Cervix cancer is a curable cancer, but achieving the best results depends on well-organized and appropriately resourced cancer services. Brachytherapy is an integral part of the cervical carcinoma treatment armamentarium. It is a technically demanding and highly specialized method of radiotherapy delivery. Depending on the equipment used, the capital expenditures and staff costs may be high. Fractionated HDR brachytherapy in the treatment of uterine cervix cancer has been increasing worldwide, including in the United States [2]. In developing countries such as Brazil, the advantages of outpatient treatment, potential cost savings, radiation protection, patient comfort, reduction of the need for general anesthesia, and less chance of applicators displacement make of this procedure an excellent treatment option [30]. Unfortunately, a well-designed prospective and randomized Phase-III trial with an adequate number of patients that would allow comparison of results between LDR and HDR brachytherapy in the treatment of cervix cancer has not yet been published. Thus, we have performed a meta-analysis to improve the statics precision of the outcomes in the clinical trials that compared these two techniques. Meta-analysis of randomized trials allows a more objective appraisal of the evidence, which may lead to the resolution of uncertainty and disagreement. It works as a valuable tool for studying rare and unintended effects of a treatment, by permitting synthesis of data and providing more stable estimates of effect.

Our results analyzing five RCTs (2,065 patients) really confirm the use of HDR as an alternative to LDR for all stages of cervical carcinoma. The overall survival and local control were similar in both groups for stages I, II and III (Figure 2 and Figure 3).

However, considering the clinical stage III a wide range of results has been found in the literature [31-36]. Vahrson and Romer [14] reported a significantly greater survival rate for stage-III patients using HDR brachytherapy. In the series of Ferrigno et al [37] stage-III patients treated with HDR brachytherapy had a poorer outcome when compared with those treated with LDR brachytherapy. Overall survival and disease-free survival at a 5-year period was statistically superior in the LDR group. These results probably are caused by the different tumor-related prognostic factors in stage-III patients, including tumor volume, extension of parametrial invasion (unilateral or bilateral), presence of hydronephrosis, lymph node metastasis, and extension of vaginal involvement. Consequently, when these patients do not receive radiotherapy combined to chemotherapy, they may still have a large volume of disease at the time of brachytherapy, even if one waits until the end of 5 weeks of daily treatment. With a large tumor volume at brachytherapy, point-A prescription simply does not cover the tumor volume. The current treatment plan and technique for gynecological brachytherapy is still based on the conventional, orthogonal film-based approach developed 40 years ago. The source loading and dose prescription of a conventional point-A plan in cervical cancer is not consistent with the individual tumor extent, resulting in either undercoverage of the tumor extent or unnecessary dosage of the surrounding normal tissue. So, in order to safely treat large volume disease (i.e. stage-IIIB patients), three dimensional (3D) image-based treatment planning is necessary to ensure proper tumor coverage. Several investigators have studied three dimensional (3D) image-based brachytherapy planning using ultrasonography, computed tomography (CT), magnetic resonance imaging (MRI), and positron-emission tomography (PET) in cervical cancer [38-46]. Although the studies had some different findings, the conventional point-A plan, compared with the 3D image-guided plan, generally overestimated the minimal dose delivered to the target volume and underestimated the maximal doses to the rectum and bladder [40,42-46]. In addition to that, 3D image-guided planning allows the evaluation of individual dose distributions applied to a certain volume, such as the gross tumor volume (GTV), clinical target volume (CTV), and organs at risk. Recently, the GEC-ESTRO working group for gynecologic brachytherapy introduced guidelines for contouring the target volumes and organs at risk (OARs) for 3D image-based treatment planning in cervical cancer [41]. It is therefore imperative with HDR for large volume disease that the practitioners contour the normal tissues and look at the dose volume values to try to minimize normal tissue dose.

Despite these limitations for large tumors (i.e. stage-IIIb and tumors > 4 cm), in recent years, HDR brachytherapy has gained popularity due to the obvious physical advantages of shortened treatment time and better geometric placement. A second major reason for conversion from LDR to HDR is reduced hospitalization. For each LDR patient of around one week of hospitalization is required, whereas, with HDR, this can be reduced to a maximum of one day. In many countries, hospitalization of patients is very expensive and methods to reduce this cost are encouraged. In others, the availability of hospital beds is a problem, especially beds in rooms suitably placed or shielded for LDR brachytherapy. There is also the problem of morbidity due to the long periods of bed-rest associated with LDR treatments. One concern with LDR intracavitary brachytherapy is the stability of positioning of the applicators during the long periods of treatment. Dose calculations are performed soon after the applicators are inserted and before they are loaded. On the few occasions that a second dosimetric study has been performed on treatment completion, this assumption has been shown to be erroneous. For example, a recent study of data from five institutions where dose distributions have been determined both at the beginning and at the end of an intracavitary application with LDR has demonstrated that 'hot-spot' dose rates to bladder and rectum increased during treatment at an average rate of 7% and 19% respectively, with negligible change in the dose rate to Point A [47].

Our results comparing late rectal and bladder complications in patients treated by HDR brachytherapy to LDR brachytherapy show that there is no difference between these two techniques. Similar probability of late complications in rectal, bladder or small intestine was observed in both groups (Table 4).

Theoretically, HDR involves a greater probability of late effects for a given level of tumor control; however, the fractionation of HDR intracavitary brachytherapy appears to offset this difference in tumor and normal tissue effects caused by an increase in dose rate.

Despite its radiobiological disadvantages mentioned by Eifel [48], the possibility of optimizing dose distribution and the lesser chance of applicator displacement seem to outweigh these disadvantages. Furthermore, the variation of dwell time with the single stepping source permits an almost infinite variation on the effective source strength and source positions, which allows for greater control of dose distribution and potentially less morbidity [25]. None of the RCTs in the literature show a higher incidence of late complications in patients with cervix cancer treated with HDR brachytherapy compared to those treated with LDR. In our meta-analysis, incidence of lower 5-year rectal complication in patients from the HDR group was probably the result of the relatively low dose delivered to the rectum with the HDR brachytherapy fractionation used. In LDR brachytherapy, the total rectal dose was commonly limited to 70 Gy. In HDR brachytherapy, this total dose was lower, depending on the fractionation used; however, how much lower remains unclear. Using the linear quadratic formula (total BED = BED *EBR *+ BED *HDR *= *nd *[1+(*d */3)] +*Br *[1+(*Br*/3)], where *n *= number of EBR fractions, *d *= dose of EBR fraction in Gy, and *Br *= total dose of HDR brachytherapy at Point A), the total dose to the rectum of 70 Gy with LDR brachytherapy corresponds to 120 Gy3 with HDR brachytherapy.

But, what is the optimal HDR fractionation schedule for treating cervical cancer? There is not a simple answer for this question. Although universally efficacious, HDR fractionation schedules cannot be ascertained, certain deductions can be made about the literature: No clear consensus of the appropriate number of fractions or the dose per fraction has been reached. Various fractionation schemes have been used "experimentally" in search of the "optimal" technique.

The GRADE system is based on a sequential assessment of the quality of evidence, followed by an assessment of the balance between benefits versus downsides, as well as the subsequent judgment about the strength of recommendations. Because frontline consumers of recommendations will be most interested in the best course of action, the GRADE system places the strength of the recommendation first, followed by the quality of the evidence. Separating the judgments regarding the quality of evidence from judgments about the strength of recommendations is a critical and specific feature of this new grading system. In our meta-analysis, the quality of evidence was moderate for mortality and local recurrence for all clinical stages, except for clinical stage I. Moreover, all included studies were RCTs with moderate percentages of follow-up. This moderate quality of evidence for mortality and local recurrence, and the low likelihood of publication bias, increase the confidence in the internal validity of our findings. Thus, our data are different of a previous and more extensive multi-institutional study including 17,068 patients treated with HDR and 5,666 with LDR at 56 institutions published by Orton et al. [49]. This involved a combination of both published data and information, collected via a questionnaire. A meta-analysis was performed on the combined data sets. The overall 5-year survival rates were similar, being 60.8% for HDR and 59.0% for LDR although, because of the large number of patients, the difference bordered on statistical significance (p < 0.045). However, since no randomization was involved, the use of p-values to demonstrate statistical significance in this context is questionable, especially with such comparable survival rates. For Stage-III patients, however, the difference in five-year survival rates was somewhat more significant, being 47.2% for HDR compared to 42.6% for LDR (p < 0.005).

The comparative results observed in our meta-analysis must be interpreted with caution, since the data comparing HDR to LDR for cancer of the cervix are fraught with bias and may be difficult to compare, due to both a lack of detailed information on the radiation administered and a wide range of external beam and intracavitary dose and fractionation schedules. Moreover, all of 5 selected studies labeled "randomized" are, in fact, not truly randomized studies and all have substantial flaws in their methodology for 'randomization'. Thus, although we have used the GRADE approach to rate the quality of evidence and strength of recommendation, the need for judgment is still required. Indeed, RCTs or meta-analysis could have important methodological differences that may impact on the results.

## Conclusion

High-dose rate brachytherapy showed comparable clinical results to LDR brachytherapy. In the subgroup analysis there is no significant difference between HDR or LDR brachytherapy considering the loco-regional recurrence, overall mortality and treatment related to late toxicities for patients with clinical stages I, II and III. Using the GRADE system, we recommend the use of HDR for all clinical stages of cervix cancer. Due to some potential disadvantages of LDR brachytherapy, such as radiation exposure of the professional staff, the need for hospitalization, the risk of anesthesia, bed immobilization that can lead to thromboembolism, discomfort of vaginal packing and applicators during bed immobilization, and displacement of the applicators, HDR brachytherapy should be considered a standard treatment strategy for patients with cervical cancer, especially in developing countries, where this procedure would have greater advantages than LDR brachytherapy. However, although a large number of fractionation schedules are in use for HDR brachytherapy, the optimal schedule has yet to be decided. Further trials are necessaries to investigate 3D brachytherapy, fractionation and dose adjustments of the total dose to reduce the frequency of complications without compromising the treatment results.

## Competing interests

The authors declare that they have no competing interests.

## Authors' contributions

GAV conceived of the study, done the statistical analysis and wrote the manuscript. GBM collected the RCTs and patient's clinical data. LIF and EJS participated in the design of the study and helped write the paper. All authors read and approved the final manuscript.
